# Electrically Controlled Bimetallic Junctions for Atomic-Scale Electronics

**DOI:** 10.1021/acs.nanolett.3c00508

**Published:** 2023-08-21

**Authors:** Anil Kumar Singh, Sudipto Chakrabarti, Ayelet Vilan, Alexander Smogunov, Oren Tal

**Affiliations:** † Department of Chemical and Biological Physics, Weizmann Institute of Science, Rehovot 7610001, Israel; ‡ Surface Physics and Material Science Division, Saha Institute of Nuclear Physics, Kolkata 700064, India; § SPEC, CEA, CNRS, Université Paris-Saclay, CEA Saclay, Gif sur Yvette 91191, France

**Keywords:** atomic contact, alloy, atomic chain, molecular junction, break junction, electromigration

## Abstract

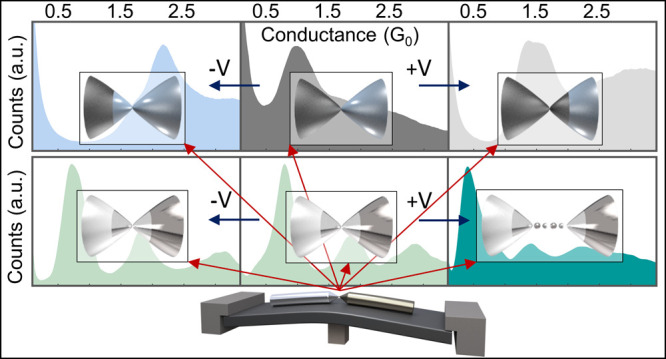

Forming atomic-scale contacts with attractive geometries and material compositions is a long-term goal of nanotechnology. Here, we show that a rich family of bimetallic atomic-contacts can be fabricated in break-junction setups. The structure and material composition of these contacts can be controlled by atomically precise electromigration, where the metal types of the electron-injecting and sink electrodes determine the type of atoms added to, or subtracted from, the contact structure. The formed bimetallic structures include, for example, platinum and aluminum electrodes bridged by an atomic chain composed of platinum and aluminum atoms as well as iron–nickel single-atom contacts that act as a spin-valve break junction without the need for sophisticated spin-valve geometries. The versatile nature of atomic contacts in bimetallic junctions and the ability to control their structure by electromigration can be used to expand the structural variety of atomic and molecular junctions and their span of properties.

Mechanically controllable break junctions (MCBJs; Figure 1a (I)) are a powerful technique, in which a contact between two metal wires is broken to form two electrodes with tips that can be brought together to make an atomic or molecular junction, when molecules are introduced between the electrodes. This technique has been used to study electronic transport at the limit of miniaturization, with the advantage of fast repeated formation and characterization of large ensembles of atomic and molecular junctions.^1−6^ Metallic MCBJs are typically based on two electrode tips made of the same metal with rare exceptions, such as the work of Scheer et al., where superconducting electrode tips (Al) bridged by a thin layer of a normal metal (Au) were used for the study of electronic transport in Au atomic contacts.^7,8^ Alternatively, bimetallic atomic contacts formed in scanning tunneling microscopes (STMs) by pressing one metal into another were reported two decades ago, revealing that a W or Ni tip indented into a Au substrate can be wetted by Au atoms.^9−12^ Although STM tip wetting by different metals has become a common practice, the early works have not been followed by further experimental studies of the structure and composition of atomic-scale contacts that are formed when two different metals are pressed together. These bimetallic structures are attractive because they can increase the structural richness of nanoscale systems in which phenomena related to electronic transport and nanomaterials can be revealed and studied.

**Figure 1:**
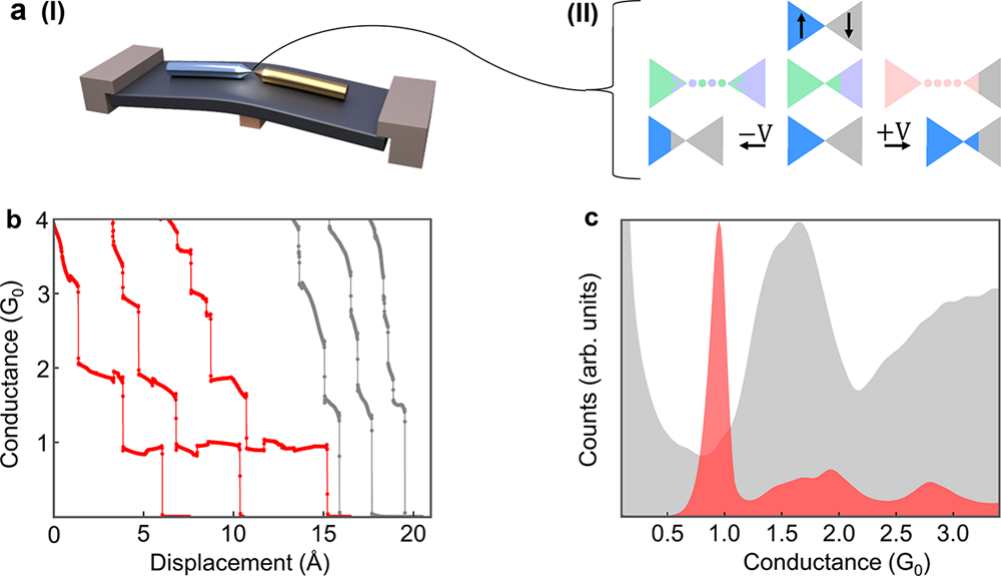
Schematics of a bimetallic break junction and conductance measurements. (a) Schematic illustration of bimetallic junctions prepared in a break-junction setup (I), and schemes (II) of an atomic-scale spin valve (top), bimetallic atomic chain (center left), metallic tip ended with other metal apex (center middle), and monometallic atomic chain suspended between different metal tips (center right) as well as atomic electromigration control over the contact composition (bottom). Each color represents a different metal type. (b) Examples for traces of conductance versus interelectrode displacement for Au–Au junctions (red) and Ni–Ni (gray) monometallic junctions. (c) Conductance histograms based on 10,000 conductance-displacement traces (as seen in b) for Au–Au and Ni–Ni junctions. The peaks indicate the most probable conductance of the atomic-scale contacts during their elongation. The measurements were done at 100 mV applied voltage.

Here, using an MCBJ setup, we find that two different metal electrodes (Au–Ni, Al–Pt, and Fe–Ni) that are pushed one against the other and then pulled back can form bimetallic atomic-scale contacts with a rich structural variety. The bimetallic structures include atomic chains composed of two types of atoms, an electrode of one metal ending with an atomic tip or even an atomic chain made of a different metal, and an atomic-scale spin valve based on repeated formation of atomic contacts between two different ferromagnets (see illustrations in Figure 1a (II)). Interestingly, the metallic composition and atomic configuration of the fabricated bimetallic structures can be modified by applying voltage pulses, where the voltage polarity dictates which type of atoms will be added or subtracted from the contact (illustrated in Figure 1a (II), bottom). We further suggest that the relative hardness of the two metals in use affects the nature of the formed structures and their response to voltage pulses. Bimetallic junctions have the potential to extend the structural variety and the wealth of properties that are demonstrated by atomic junctions, as well as by molecular junctions, since the latter can be formed by introducing molecules into bimetallic junctions.

The goal of this Communication is to report on the structural versatility of atomic contacts formed in bimetallic junctions and their tunability thanks to atomically precise electromigration. Combining the properties of different metals in a single atomic-scale structure opens the door for the use of these systems as an advanced testbed for the study of charge, spin, and heat transport as well as material properties, including proximity effects in atomic structures, nanoscale electromigration, and atomic-scale alloying. Our findings raise questions related to structural, mechanical, and electronic properties of bimetallic atomic contacts that will not be addressed here and call for follow-up studies.

The studied bimetallic contacts are prepared in MCBJ setups (Figure 1a (I)). A flexible substrate is first bent. Then, two wire segments (electrodes) made of different metals, each with a sharp tip, are attached to the bent substrate, with their tips pointing to each other. Next, the substrate is relaxed to its flat configuration, and the tips are squeezed against each other to form a macroscale contact. This break junction is placed in a vacuum chamber and cooled to 4.2 K. To prepare an atomic-scale contact, the substrate is bent at its center by a piezoelement that pushes it against two stoppers. Consequently, the tips are pulled apart, and the contact cross-section is gradually reduced until a contact with a single-atom diameter is formed between the electrodes. Further stretching breaks the junction. A new atomic contact can be prepared by relaxing the substrate, such that the electrode tips are pushed against each other to have a multiatomic contact, then the electrodes are pulled apart to reform a single-atom contact. This process can be repeated thousands of times to study ensembles of junctions with different atomic-scale configurations. During the repeated rupture-formation process, the junction’s conductance (current/voltage) is recorded as a function of interelectrode distance (Supporting Information, section 1). The repeated squeezing and stretching promote junction cleaning from adsorbed contamination on the initially prepared tips. This is verified by conductance analysis, as detailed below, and in Supporting Information, section 2.

Starting with homometallic junctions, where a single wire is broken and reformed in cryogenic vacuum, Figure 1b presents conductance traces as a function of interelectrode displacement during stretching of Au–Au (red) and Ni–Ni (gray) junctions. The conductance drops in steps whenever the contact diameter between the electrodes is reduced, and the last plateau before junction rupture indicates the conductance of a single-atom contact.^13−18^ Since the conductance characteristics can vary between different contact realizations, Figure 1c shows conductance histograms, based on 10,000 conductance traces, each with peaks that identify the most probable conductance during junction stretching. The repeated plateaus seen in Figure 1b for single-atom contacts at ∼1 *G*_0_ for Au–Au junctions and ∼1.6 *G*_0_ (sometimes also at ∼1.2 *G*_0_(16−20)) for Ni–Ni junctions construct dominant peaks in the respective Figure 1c histograms (*G*_0_ ≅1/12.9 (kΩ)^−1^ is the conductance quantum). The ∼1 *G*_0_ conductance of single Au–Au atomic contacts is mostly given by an almost fully open conduction channel, dominated by the s valence orbitals of Au.^7,21^ The higher conductance of single Ni–Ni atomic contacts comes from several partially open conduction channels, associated with s, p, and d valence orbitals.^22−24^ Other features seen at higher conductance are related to multiatomic contacts. The different shapes of the conductance histograms seen in Figure 1c can therefore be used to distinguish between the formation of Au–Au and Ni–Ni atomic-scale contacts.

We now turn to examine bimetallic junctions based on three metal pairs, Au–Ni, Al–Pt, and Fe–Ni, which are different in their relative hardness (Au ≪ Ni, Al < Pt, Fe≅Ni,^25^ see Supporting Information, Table S1). Within each pair, each metal forms junctions (e.g., Au–Au or Ni–Ni junctions in the first pair) that have a different histogram shape (e.g., Figure 1c). Figure 2a I–III presents a typical histogram for Au–Ni bimetallic junctions, together with histograms for Au–Au and Ni–Ni junctions for convenient comparison. The Au–Ni histogram is essentially identical to the Au–Au histogram, indicating that although the two electrodes are made of different metals, the atomic-scale constriction of the junction that dominates its conductance is made of Au (illustrated in Figure 2a II, inset). The formation of pure Au atomic chains during the stretching of Au–Ni junctions with a typical Au–Au interatomic distance further supports this conclusion (see details below). Note that Egle et al. produced by lithographic processing Au–Co–Au and Co–Au–Co break junctions with a central Co or Au section between the outer Au or Co electrodes, respectively. They report the formation of Co–Co and Au–Au contacts in the first case and Au–Au contacts in the second case when the junction is broken and reformed, perhaps consistent with a preference for Au wetting of a harder metal as Co.^26^Figure 2b I–III reveals that the histogram of Al–Pt junctions is similar to that of Al–Al junctions but differs from that of Pt–Pt junctions. Therefore, for Al–Pt junctions, the Pt electrode is covered with Al atoms, forming a monometallic Al contact (illustrated in Figure 2b II, inset). In contrast to the mentioned two cases, where one metal wets the other, Figure 2c I–III shows that the histogram of Fe–Ni significantly differs from that of Fe–Fe and Ni–Ni junctions, indicating the formation of atomic contacts that contain both metals (illustrated in Figure 2c II, inset). Our calculations in Supporting Information section 3 ascribe the lower conductance of the main peak in the Fe–Ni histogram (∼1.0 *G*_0_) with respect to the main peaks of the Fe–Fe and Ni–Ni histograms to an abrupt Fe–Ni atomic contact or the formation of an alloy in the contact. For the Fe–Ni junction, the comparable hardness of the two metals most likely suppresses a dominant wetting of one metal tip by the other metal, whereas for Au–Ni and Al–Pt junctions, the softer metal (Au and Al, respectively) constructs the atomic contact by wetting the harder metal (Ni and Pt, respectively).

**Figure 2:**
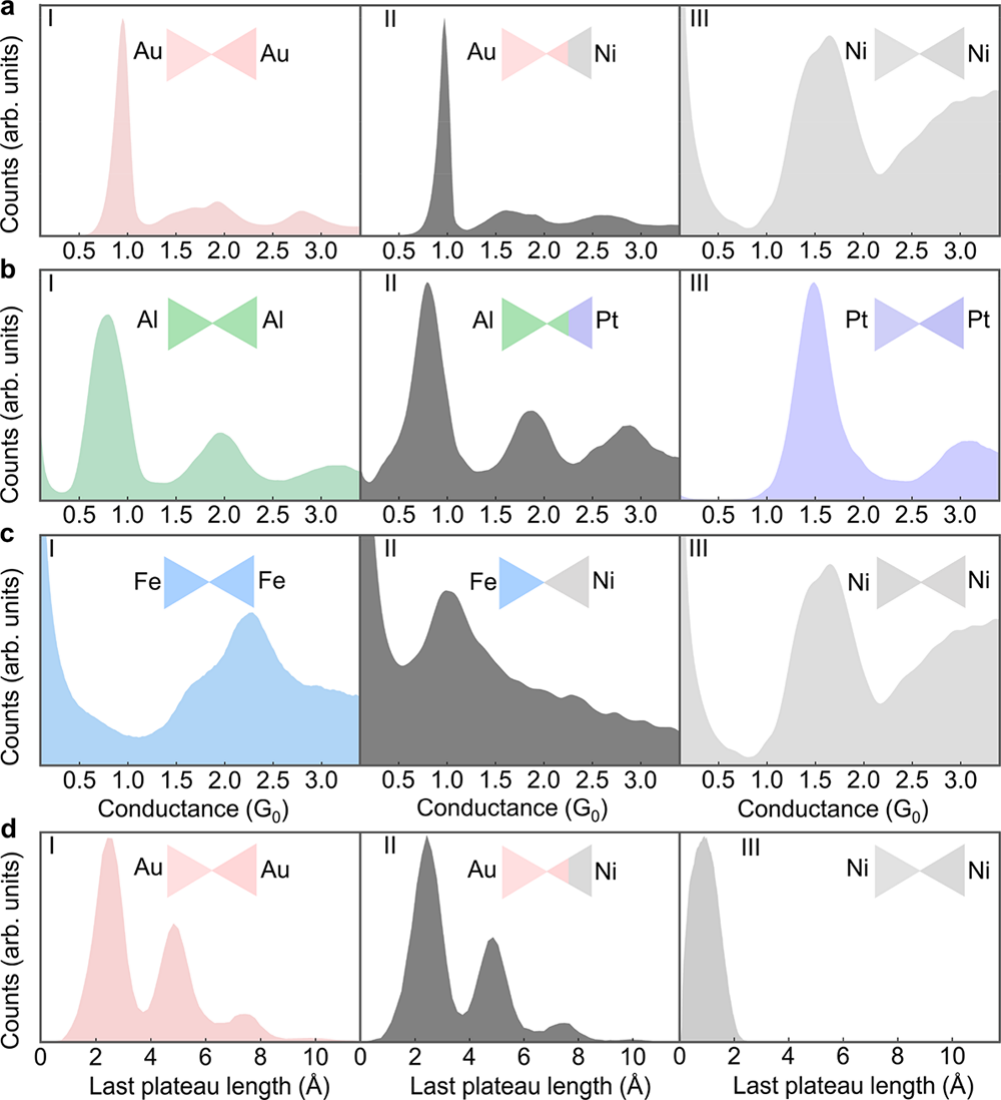
Conductance and length histograms of monometallic and bimetallic atomic-scale junctions. (a) Conductance histograms of Au–Au (I), Au–Ni (II), and Ni–Ni (III) junctions. (b) Conductance histograms of Al–Al (I), Al–Pt (II), and Pt–Pt (III) junctions. (c) Conductance histograms of Fe–Fe (I), Fe–Ni (II), and Ni–Ni (III) junctions. The insets schematically illustrate the metallic composition of the formed atomic contacts in view of the histograms and the described analysis in the text. (d) Length histograms of Au–Au (I), Au–Ni (II), and Ni–Ni atomic-scale junctions. The average interpeak distance between the first three peaks in I and II is 2.5 ± 0.2 and 2.5 ± 0.3 Å, respectively. Each histogram is based on 10,000 conductance-displacement traces, taken during junction elongation at 100 mV applied voltage. Length histograms consider data in the main conductance peak range (0.7–1.1 *G*_0_ for I and II and 1.0–2.0 *G*_0_ for III).

Coming back to the case of Au–Ni atomic junctions, while it is known that elongation of Au–Au junctions can form suspended atomic chains between the electrodes,^14^ elongating Ni–Ni junctions does not form such chains. Figure 1b shows that the last ∼1 *G*_0_ conductance plateau that is associated with the elongation of a Au–Au single-atom contact can have different lengths. Collecting the number of times that a certain ∼1 *G*_0_ plateau length was found in 10,000 traces gives the length histogram presented in Figure 2d (I). This histogram reflects the relative probability of finding an atomic contact with a certain length. The set of peaks is a known fingerprint for the formation of atomic chains with a different number of atoms, whereas the average distance between the peaks is a good measure for the average Au–Au interatomic distance in the elongated chain.^27−29^ Here, it is 2.5 ± 0.2 Å for the first three peaks. Interestingly, the elongation of Au–Ni atomic-scale junctions produces an identical length histogram (Figure 2d (II)), with an average interpeak distance between the first three peaks of 2.5 ± 0.3 Å. This indicates that chains made of Au atoms with no Ni or other contaminants are formed in the junctions. Note that the incorporation of foreign atoms (e.g., oxygen) in atomic chains alters the interpeak distances.^20,30^ Finally, Figure 2d (III) shows that Ni–Ni does not form atomic chains. As mentioned, wetting a metal tip with Au is a known process. However, the formation of suspended Au atomic chains attached to a different metal, Ni in our case, is an unknown structure. This structure can be used, for example, for the study of magnetic proximity effects in Au atomic chains.

The application of a voltage pulse of +1 or −1 V for 200 μs allows control over the structure and composition of the studied bimetallic atomic contacts and even promotes the formation of new structures. Here as well, we suggest that the relative hardness of the metals may play an important role. As shown in Figure 3a, the application of a voltage pulse does not alter the histograms of Au–Ni junctions. However, repeating the procedure for Al–Pt junctions (Figure 3b) leads to interesting behavior. When the pulse is negative, as when electrons are injected from the Al electrode, no change in the histogram is seen. Following a positive voltage pulse, where the electrons are injected from the Pt electrode, the histogram is clearly modified, having a dominant peak at ∼0.45 *G*_0_ that is lower than the conductance of a single-atom contact in Al–Al or Pt–Pt junctions. The distinct conductance indicates the formation of atomic-scale contacts that involve both Pt and Al atoms. As will be shown, this contact can be elongated to an atomic chain made of Al and Pt atoms. Our calculations in the Supporting Information, section 3, further relate the low conductance to chain formation.

**Figure 3:**
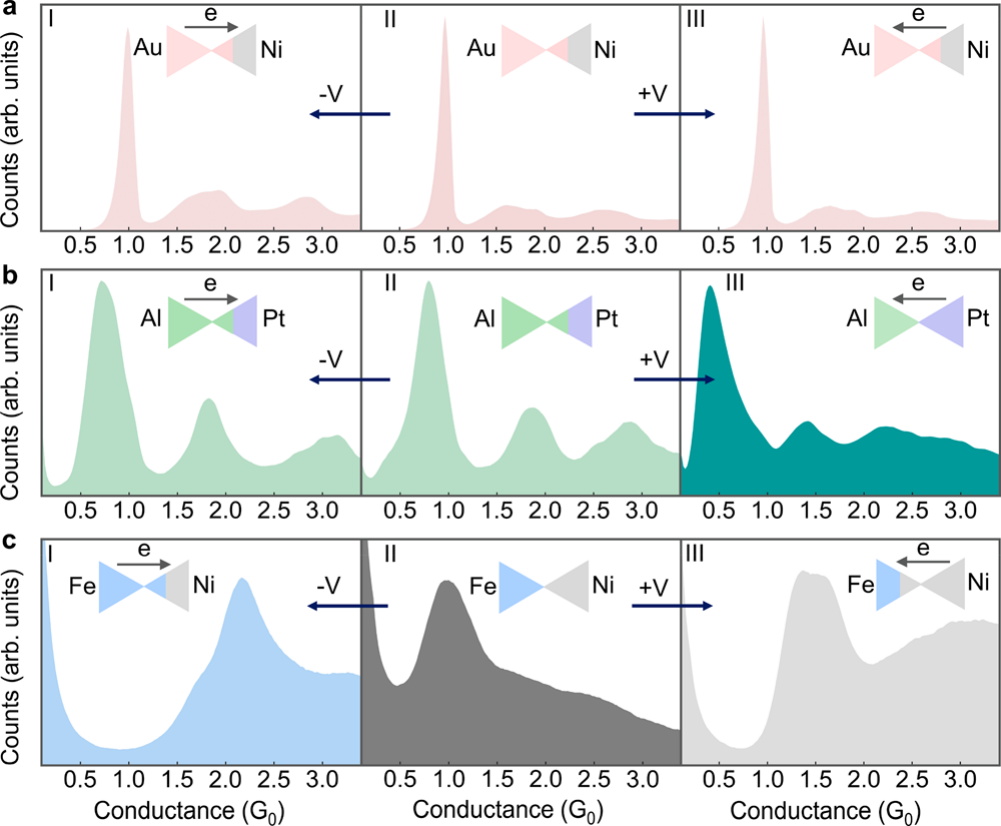
Conductance histograms of bimetallic atomic-scale junctions before and after the applied voltage pulse. (a) Conductance histogram of Au–Ni atomic-scale junctions, after the application of a negative voltage pulse (I), before the application of a voltage pulse (II), and after the application of a positive voltage pulse (III). (b) Conductance histogram of Al–Pt atomic-scale junctions, after the application of a negative voltage pulse (I), before the application of a voltage pulse (II), and after the application of a positive voltage pulse (III). (c) Conductance histogram of Fe–Ni atomic-scale junctions, after the application of a negative voltage pulse (I), before the application of a voltage pulse (II), and after the application of a positive voltage pulse (III). The voltage pulse magnitude is 1 V, and it is applied for 200 μs to a junction with a 3 *G*_0_ conductance (preadjusted by changing the inter electrode distance) before taking the histograms. Each histogram is based on 10,000 conductance-interelectrode displacement traces, taken during junction elongation at an applied voltage of 100 mV.

Figure 3c presents Fe–Ni histograms before and after the application of a voltage pulse. Both positive and negative pulses led to clear histogram modifications. When electrons are injected from the Ni electrode (+1 V pulse), the resulting histogram is similar to that of a Ni–Ni junction. Namely, the atomic Fe–Ni contact was replaced by a Ni–Ni contact (the Fe electrode ends with a tip of Ni atoms). When electrons are injected from the Fe electrode (−1 V pulse), the obtained histogram is similar to that of an Fe–Fe junction. The conductance histograms of pure Ni–Ni and Fe–Fe junctions have a typical main peak with a shoulder at the low conductance side (e.g., Figure 2c (I, III)). In both cases, this shape is ascribed to two or more populations of atomic contacts with different structures, and therefore somewhat different conductance is expected.^16,18,31^ In view of Figure 3, the voltage pulse effectiveness seems to be related to the relative hardness of the two metals, although other properties such as surface energy, intermetallic bond strength, and the tendency for alloying may also play a role. For Au–Ni junctions, the applied pulse could not overcome the strong tendency of Ni wetting by Au, whereas for Fe–Ni electromigration of atoms from both electrodes was observed. In the “intermediate” case of Al–Pt junctions, a pulse that involves electron injection from the Pt electrode could introduce Pt atoms into the junction, though a pure Pt contact could not be obtained. The directionality of the induced atomic rearrangement can be nicely identified thanks to the two different metals used as electrodes. Thus, bimetallic break junctions can be an interesting system for the study of atomic-scale electromigration.

We now focus on bimetallic atomic contacts formed after applying a positive voltage pulse to the Al–Pt junctions. This procedure yielded the conductance histogram shown in Figure 3b (III). Figure 4a presents a length histogram of the last conductance plateaus measured during the elongation of Pt–Pt junctions. As mentioned before, a set of peaks in a length histogram indicates atomic-chain formation,^14,27−29,32−34^ where the peaks reflect the relative probability for chains with a different number of atoms. Al–Al junctions, however, do not form atomic chains. Once a single Al atom contact is formed, it simply breaks when stretched without pulling atoms from the electrodes, though Al atomic dimers can be formed with a low probability. This is manifested by a shorter length histogram for Al–Al junctions (Figure 4b), with one main peak and a minor shoulder due to dimer formation. Al–Pt junctions produce length histograms similar to those of Al–Al junctions (Figure 4c). However, following the application of a positive voltage pulse with electrons injected from the Pt electrode, the length histogram changes considerably, as seen in Figure 4d. Here, the length distribution is clearly longer than expected for a single-atom contact. This behavior can be ascribed to the formation of atomic chains, despite the absence of well-separated peaks.^20^ The lack of distinct peaks may indicate that the elongated chains are not exclusively made of Pt atoms. The histogram smearing can stem from atomic chains that contain different numbers of Pt and Al atoms and their permutations, leading to a large variety of chain lengths. Therefore, the data presented in Figure 4d and Figure 3b (III) suggest the formation of bimetallic atomic chains with lower conductance than that of Al–Al and Pt–Pt atomic junctions (see also calculations in Supporting Information, section 3). In Supporting Information, section 5, we suggest a chemical indication for the presence of Al atoms within the atomic chains. The indication is based on the conductance signature of Al atoms in elongated contacts that stems from the response of electronic states related to Al sp_*z*_ orbitals to interatomic stretching.^35−40^ To date, the formation of suspended bimetallic atomic chains has been demonstrated only in atomic-scale constrictions in metal alloys, as reported for AuAg, AuCu, and PtIr alloys.^41−43^ Here, we show that bimetallic atomic chains can be formed at the contact between two different metals (see Supporting Information, section 6).

**Figure 4:**
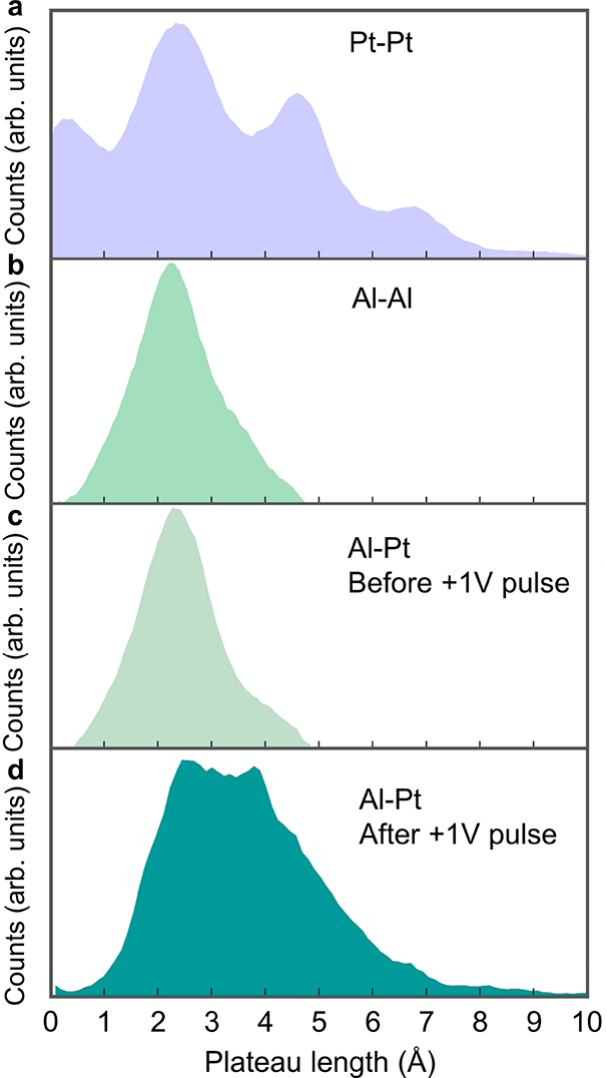
Length histograms of monometallic and bimetallic atomic-scale junctions. (a) Length histogram of Pt–Pt atomic-scale junctions. (b) Length histogram of Al–Al atomic-scale junctions. (c) Length histogram of Al–Pt atomic-scale junctions before the application of a voltage pulse. (d) Length histogram of Al–Pt atomic-scale junctions after the application of a positive voltage pulse (+1 V for 200 μs; electrons are injected from the Pt electrode to a 3 *G*_0_ Al–Pt contact). Each histogram is based on 10,000 conductance vs interelectrode displacement traces taken during junction elongation at an applied voltage of 100 mV. Length histograms consider data in the conductance range of the main peak in the conductance histograms: (a) 1.0–2.5 *G*_0_, (b) 0.3–1.3 *G*_0_, (c) 0.3–1.3 *G*_0_, and (d) 0.1–1.1 *G*_0_ (see the mentioned conductance histograms in Figures 2 and 3).

To exemplify the potential of bimetallic junctions, we present an Fe–Ni spin-valve break junction. Although atomic and molecular spin-valve junctions have been demonstrated in break-junction setups (e.g., refs (44−46)), these structures typically require nontrivial nanofabrication to promote magnetization switching in each electrode at a different applied magnetic field. Furthermore, careful design and challenging fabrication are required to minimize magnetostriction that leads to conductance variations due to changes in the interelectrode distance when the magnetization changes. Here, we utilize the different coercive fields of Fe and Ni to flip the magnetization of each electrode separately in our Fe–Ni break junctions while collecting data on thousands of junctions. Figure 5 shows the most probable conductance of a single-atom contact in Fe–Ni junctions as a function of magnetic fields perpendicular to the junction axis. At each magnetic field, several conductance histograms were taken consequentially, and the average conductance value of the main peak in these histograms is presented in Figure 5a. Examples of conductance histograms taken under different fields for parallel and antiparallel electrode magnetizations are seen in Figure 5b,c (see also the Supporting Information, section 7). The conductance is high for a parallel magnetization, and it is lower for an antiparallel magnetization. Between 0.2 and 0.4 T (or −0.2 and −0.4 T), where T denotes Tesla, the field is high enough to flip the magnetization in the Ni electrode (Ni has a lower coercive field than Fe), while the magnetization direction of the Fe electrode is preserved. However, above this field range, the magnetization of the Fe electrode flips as well, yielding a parallel magnetization. The obtained magnetoresistance (MR) of 7.6 ± 0.7% is typical for atomic spin valves of ferromagnetic metals^44^ (MR = (*G*_P_ – *G*_AP_)/(*G*_P_ + *G*_AP_), where *G*_P_ and *G*_AP_ are the conductance for parallel and antiparallel configurations, respectively). This bimetallic structure can therefore serve as a platform for molecular-junction spin valves. Using bimetallic break junctions as a spin valve has the following advantages: (i) complicated nanofabrication is not required; (ii) avoidance of magnetostriction artifacts, since conductance histograms are collected at fixed magnetic fields and magnetizations; (iii) magnetoresistance of thousands of junctions can be collected in a short time; and (iv) high mechanical stability is preserved. Thus, a spin-valve break junction can be used as a convenient setup for the study of spin transport and magnetism in atomic or molecular junctions.

**Figure 5:**
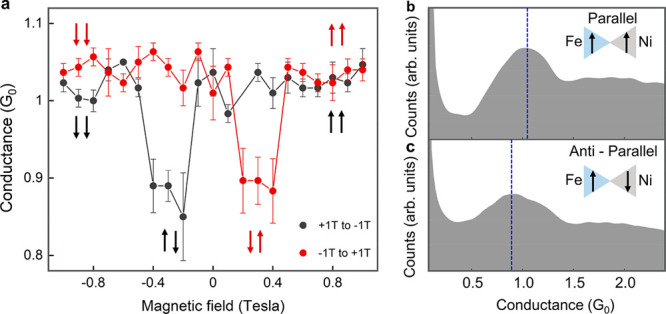
Mechanically controllable spin-valve junction. (a) Most probable conductance of Fe–Ni atomic junction as a function of applied magnetic fields perpendicular to the junction axis (T denotes Tesla). The data at each magnetic field are obtained from at least 5 consecutive conductance histograms. Each histogram is based on 10,000 conductance traces measured during junction elongation, at a bias voltage of 100 mV. The error bars provide the standard deviation of the averaged data. (b, c) Conductance histograms taken at magnetic fields of −1 T (b) and −0.2 T (c), yielding a relative high and low most probable conductance (blue dashed lines), ascribed to parallel and antiparallel magnetization as illustrated in the insets, respectively.

The rich structures of atomic-scale contacts formed when two different metals are repeatedly pressed can provide a versatile platform for scientific research. For example, bimetallic junctions can serve as a natural testbed for the study of alloys with reduced dimensions, bimetallic atomic interfaces, electromigration at the atomic scale, and proximity effects related to atomic structures near superconducting or ferromagnetic electrodes. The compositions and geometries of bimetallic atomic contacts are also attractive for the study of spin, charge, and heat transport at the atomic scale. Furthermore, the introduction of molecules to bimetallic junctions can merge the structural advantages of these junctions and that of molecules to gain a wealth of new properties and functionalities.

## Supporting Information Available

Experimental details, control experiments, table of metal hardness, recognition of Al atoms in atomic-chains formed in Al–Pt junctions, Al–Pt junction response to repeated deformation cycles after pulse application, density functional theory and transport calculations, including technical calculation details (PDF)
