# Supplementary material for: Electrically Controlled Bimetallic Junctions for Atomic-Scale Electronics
Source: Nano Lett. 2023 Aug 21;23(17):7775–81. doi: 10.1021/acs.nanolett.3c00508 (PMC10510575; doi:10.1021/acs.nanolett.3c00508)
Supplement: Supplementary file 1 — nl3c00508_si_001.pdf [file nl3c00508_si_001.pdf]

Supporting Information

Electrically-controlled bimetallic junctions for atomic-scale electronics

Anil Kumar Singh[1], Sudipto Chakrabarti[1,2], Ayelet Vilan[1], Alexander Smogunov[3], and Oren Tal[1,*]

[1]*Department of Chemical and Biological Physics, Weizmann Institute of Science, Rehovot 7610001, Israel*

[2]*Surface Physics and Material Science Division, Saha Institute of Nuclear Physics, Kolkata 700064, India*

[3]*SPEC, CEA, CNRS, Université Paris-Saclay, CEA Saclay, Gif sur Yvette 91191, France*

* Corresponding author

Content:

Section 1: Experimental details

Section 2: Control Experiments with monometallic junctions

Section 3: Density functional theory and transport calculations

Section 4: Supplementary table of metal hardness

Section 5: Recognition of Al atoms in atomic chains formed in Al-Pt junctions

Section 6: Al-Pt junction response to repeated deformation cycles after pulse application

Section 7: Spin-valve experiments

Section 1: Experimental details

We use a mechanical controllable break-junction set-up[S1] to fabricate the study bimetallic atomic scale structures (Figure 1a, main text). The break junction samples are composed of two wires (supplier: Alfa Aesar, diameter: 0.1 mm, length 12 mm) each made of a different metal, with the following purities: 99.994% (Ni), 99.998% (Au), 99.997% (Pt) 99.999% (Al), and 99.998% (Fe). First, the two wires are cut to have a sharp tip at one end. Then, as illustrated in Figure 1, in the main text, a flexible substrate is temporarily bent in the middle (1-mm-thick phosphor-bronze plate covered by 100 μm insulating Kapton film), and the wires are attached to the substrate such that their tips are in contact. Finally, the bent substrate is relaxed, such that the flexible substrate is straitened, and the two metal tips are squeezed into each other to form a larger contact between the two tips. Samples, with the same metal electrodes are formed by attaching a single wire (24 mm in length) with a notch (partial cut) at its center to the flexible substrate.

The sample is placed in a vacuum chamber that is pumped and cooled to ~4.2K. With the aid of a three-point bending mechanism including a piezoelectric element (PI P-882 PICMA) connected to a Piezomechanik SVR 150/1 piezo driver, which is driven by a 24-bit NI-PCI4461 or a NI-PXI4461 data acquisition (DAQ) card, the substrate is bent in cryogenic vacuum conditions to either break the contact between the wires for bimetallic junctions or break the wire at the notch for monometallic junctions. Since the three-points bending mechanism translates the movement of the piezoelectric element to more than two orders of magnitude attenuated interelectrode displacement, the distance between the electrode apices can be controlled in sub-Ångstrom resolution. Stretching the contact/notch reduces the number of atoms in the cross section of the constriction down to a single atom, where further stretching leads to junction rapture into two atomically sharp apices. To promote the formation of new structures at the contact and collect information on the span of possible formed structures at the contact, the junction can be repeatedly broken and reformed (typically up to conductance of 70 G_0) at a rate of 20–40 Hz, while the conductance of the junction is measured simultaneously via the two wire segments that serve as electrodes.

To measure conductance, the junction is biased with a d.c. voltage provided by the NI-PCI4461 or NI-PXI4461 DAQ card. The presented measurements are performed at a bias voltage of 100 mV (1,000 mV is applied by the DAQ card, via a 1/10 voltage divider to improve the signal to noise

ratio). The resulting current from the junction is amplified by a current amplifier (Femto amplifier DLPCA 200) and recorded by the DAQ card. To extract the conductance, the obtained current values are divided by the applied voltage values. The interelectrode displacement is found by the exponential dependence of tunneling currents on the separation between the electrodes[S2].

Histogram formation: Conductance histograms were formed using 200 bins with a bin width of 0.02 G_0 (linear histograms). Length histogram were constructed using dedicated MatLab code. In short, the code counts the number (n_t) of readings (G_i), within a specific conductance window ($[G_{min} \quad G_{max}]$) for each pull trace: $n_t = \sum G_{min} \leq G_i \leq G_{max}$; the net length per trace (L_t) is defined as: $L_t = \kappa \cdot \Delta V_p \cdot (n_t - 1)$, where κ is the break-junction spring constant deduced from the tunneling-decay slope and ΔV_p is the increment in piezo-voltage. Finally, a histogram is made by MatLab standard function: *histcounts.m* using as input the ~10,000-long array of L_t values deduced for the entire dataset (no selection).

Application of a voltage pulse: Figures 2aII, 2bII, and 2cII in the main text present typical conductance histograms of the relevant bimetallic junctions before the application of a voltage pulse. Subsequently, we formed a junction with 3 G_0 conductance and applied a single +1 V or a -1 V voltage pulse for 200 μs. Following the single pulse application, we recorded another conductance histogram, such as Figures 2aI or 2aIII, 2bI or 2bIII, and 2cI or 2cIII. To restore the initial conductance histograms, we repeatedly broke and reformed the junction, where during the formation we reached ~70 G_0 to promote junction deformation. This process was applied for 35,000 cycles for all three bimetallic junctions after a pulse procedure, even when no apparent change in the conductance histogram was seen (e.g., in the case of Au-Ni). Following the described procedure, and after ensuring a successful reconstruction of a conductance histogram identical to the initial one, another pulse was given and the procedure was repeated.

Section 2: Control Experiments with monometallic junctions

Out of the monometallic junctions based on Au, Ni, Al, Pt, and Fe that are used in this work, the shape of the conductance histograms of Ni and Pt junctions are the most sensitive to contaminations within the junction. Therefore, we selected these two metals as testbeds in the following experiments. We fabricated Ni-Ni and Pt-Pt break junction samples based on two sharp electrodes prepared in ambient conditions using an identical procedure to the one used for the bimetallic break junctions (as described in Section 1). Figure S1 shows that the conductance histograms of these junctions are identical to the ones of Ni-Ni and Pt-Pt break junctions fabricated using a single wire (made of Ni or Pt, respectively) that was broken only after cryogenic conditions were achieved. Interestingly, the presented histograms in Figures S1a,b were recorded after 1,000 initial repeated breaking and formation cycles, indicating that the removal of possible contaminations does not require considerable efforts.

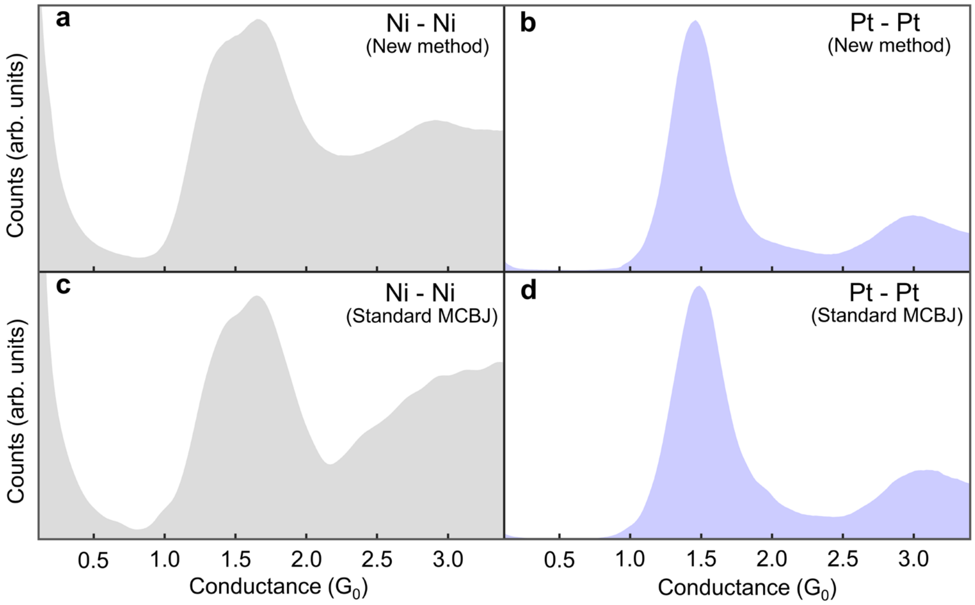

Figure S1: (a,b) Conductance histograms of Ni-Ni (a) and Pt-Pt (b) atomic junctions prepared using the new MCBJ preparation method described in Section 1. (c,d) Conductance histograms of Ni-Ni (b) and Pt-Pt (c) atomic junctions prepared using the standard MCBJ preparation method described in Section 1. The peaks indicate the most probable conductance of the atomic scale contacts during their elongation. The measurements were done at 100 mV applied voltage, and the histograms are composed of 10,000 conductance traces each.

Section 3: Density functional theory and transport calculations

Figure S2 presents the calculated transmission for junctions with different metallic compositions, where the transmission at the Fermi energy provides the conductance in units of G_0. The associated projected density of states (s and p for Al, s and d for Pt, Ni and Fe) on the central apex atoms are presented we well.

Bellow, we list relevant information about the calculations:

1. To overcome the technical limitations involved with calculations of contacts between two different metal electrodes, we used the same metal for the two electrodes, with different metal atoms at the contact, as illustrated in Figure S2, top insets. This is done under the assumption that the transmission properties of the atomic junctions are dominated by the atoms at the contact vicinity.

2. For Ni-Ni and Ni-Fe junctions, we present in Figure S2d the spin resolved PDOS. However, the presented transmission in Figure S2b provides the sum of spin up and down contributions, since we are interested in comparing the calculations to the measured total (spin up and down) conductance.

3. Pt-Pt and Fe-Fe atomic junctions have a higher measured conductance than Al-Al and Ni-Ni junctions, respectively. Here, we do not present calculations for these junctions. Instead, we focus on a comparative analysis between the transmission of the less conducting Al-Al and Ni-Ni counterpart junctions with respect to their relevant bimetallic systems Al-Pt and Ni-Fe, respectively.

4. When introducing Pt atoms to the Al hosting structure to form an abrupt Al-Pt atomic contact, the two atoms tend to mix at their back multi-atomic interface (Figure S2a, red frame Inset, inside the right electrode apex) after structural relaxation. This is in contrast to the case of an abrupt Ni-Fe contact, where the initially well-separated Fe and Ni atomic sub-systems are kept separated after structural relaxation (Figure S2b red frame Inset, inside the right electrode apex).

5. When considering an alloy contact at the junction constriction, we locally form an alloy made of a similar number of atoms of each metal as a representative composition. More elaborated calculations for different alloy compositions in the junctions are beyond the scope of this Letter.

Focusing on the calculations outcome, the calculated ∼0.9 G_0 transmission for an Al-Al atomic junction (Figure S2a; black) at the Fermi energy is very close to the measured conductance for Al-Al atomic junctions, as obtained by conductance histograms (e.g., Figure 1bI, main peak around 0.8 G_0). Conductance histograms obtained for Ni-Ni junctions reveal two main most probable conductance values at ∼1.6 G_0 and sometimes also at ∼ 1.2 G_0 (e.g., Figure 2cIII), ascribed to two dominant stable configurations (Refs.16-20 in the main text). Here, the calculated transmission (Figure S2b; black) at the Fermi energy agrees well with the measured ∼1.6 G_0 conductance.

Figure S2a shows that an abrupt contact between Al and Pt (red) has a higher transmission than the Al-Al junction (black). Interestingly, even an alloy contact with a short chain shows a higher transmission compared to the Al-Al atomic junction (blue). Note that we first produced a single atom contact alloy but following relaxation a short atomic chain was formed, indicating that such a chain is energetically favorable. In contrast to the mentioned cases, a lower calculated transmission is clearly observed for an Al-Pt alloy at the contact with a longer atomic chain that adapt (to some extent) a zigzag configuration. This structure was formed by increasing the interelectrode separation in the former structure, following by relaxation. This process yielded the longer chain, again showing the preference for Al-Pt bimetallic chain formation. Note that the elongation of the chain leads to PDOS reduction on the Pt central atom, which we associate with the observed reduction in transmission. To summarize, an abrupt Al-Pt atomic contact cannot explain our experimental observations, and the same applies to the considered alloy Al-Pt junction. Conversely, an alloy junction with a longer atomic chain does show a clear reduction in transmission with a value at the Fermi energy (~ 0.64) closer to the observed conductance after the application of a positive voltage pulse (Figure 3bIII, main peak centered at ~ 0.45 G_0 with a tail towards a higher conductance). This is consistent with experimental indications for the elongation of bimetallic Al-Pt atomic chains following the application of a +1V pulse (before pulse: Figure 4c; after pulse: Figure 4d), alongside with a reduction in conductance (before pulse: Figure 3bII; after pulse: Figure 3bIII).

Figure S2b shows that a junction with an abrupt bimetallic Fe-Ni contact, and a Fe/Ni alloy contact have a lower transmission at the Fermi energy than that of a Ni-Ni atomic junction. For an abrupt Ni-Fe junction the transmission has a dip around the Fermi energy. As a result, the conductance is close to 1 G_0. The PDOS analysis in Figure S2d allows attributing this dip to a significant depletion

of spin down d-states at the Fermi energy on the Ni apex atom, driven by hybridization between the Ni and Fe apex atoms. A similar effect is also seen for the alloy Ni-Fe junction, even within a wider energy window around the Fermi energy. To summarize, in view of the calculations the lower measured conductance for Ni-Fe junctions in comparison to Ni-Ni junctions can be ascribed to the formation of either an abrupt bimetallic contact or an alloy at the junction's constriction.

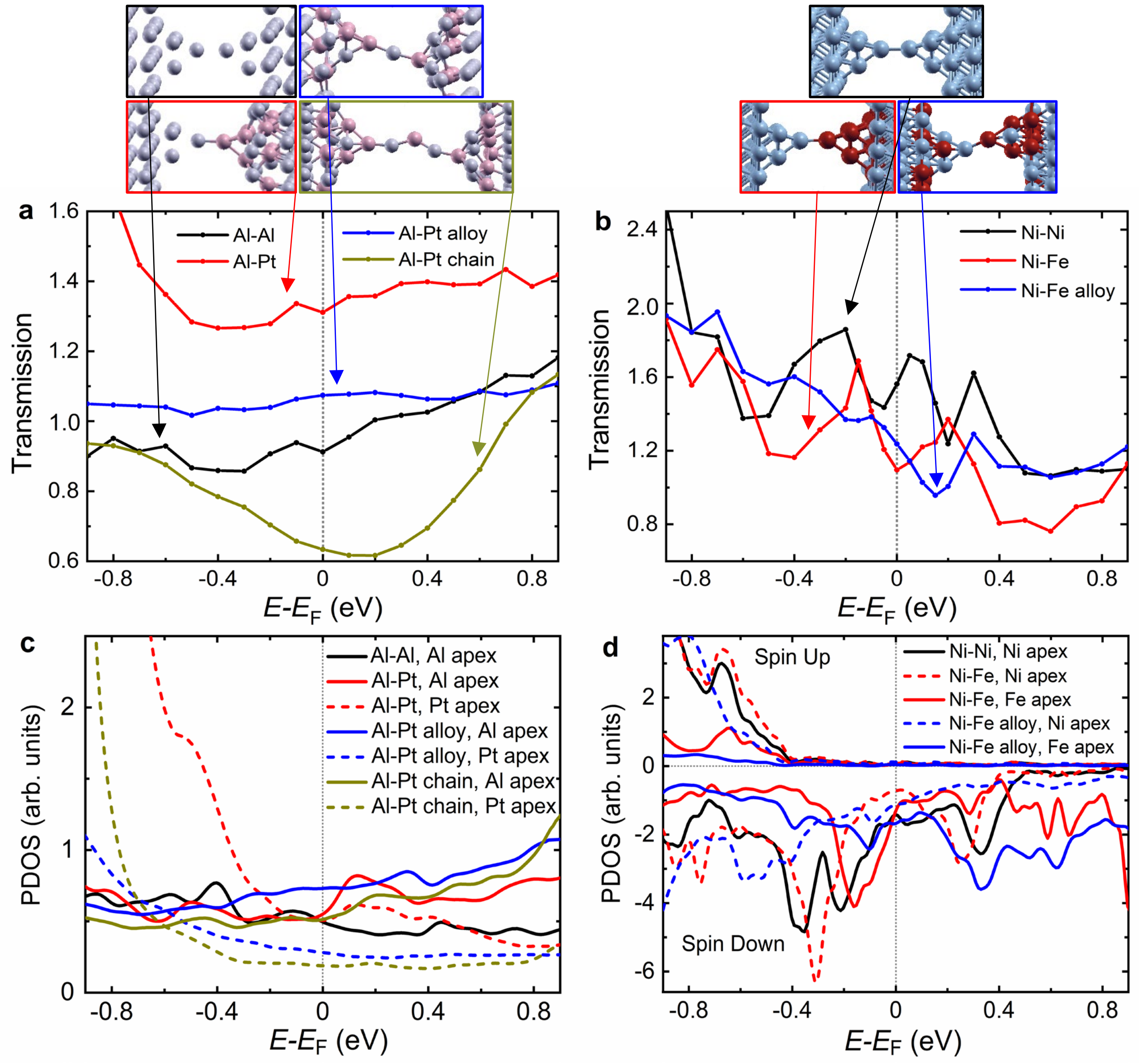

Figure S2: Calculated transmission and projected density of states (PDOS) for atomic junctions. (a) Transmission of Al-Al and different Al-Pt atomic junctions. (b) Transmission of Ni-Ni and different Ni-Fe atomic junctions. (c) PDOS of Al-Al and different Al-Pt atomic junctions. (d) Spin resolved PDOS of Ni-Ni and different Ni-Fe atomic junctions. In all cases the projection is done on the most central atoms.

Technical details:

In order to interpret the experimental results, we performed density functional theory (DFT) calculations using Quantum-ESPRESSO (QE)[S3] package with Perdew-Burke-Ernzerhof (PBE)[S4] parametrization for exchange-correlation functionals. We concentrated on several structures of Al,Pt and Ni,Fe based junctions. The atomic junctions were simulated by supercells consisting of three (left) and four (right) Al or Ni atomic layers in the (111) crystallographic orientation connected by two 4-atoms pyramids as shown in Figure S2. A (4x4) in-plane periodicity (16 atoms per layer) was employed to avoid artificial interactions between junctions. The energy cut-offs of 30 and 300 Ry were used for the wave functions and charge density expansions over plane waves, while electron-ions interactions were described by ultra-soft pseudopotentials. A (4x4x1) k-mesh was adopted with a smearing parameter of 0.01 Ry in order to integrate over the Brillouin zone.

In order to simulate bi-metallic junctions, the composition of the two pyramids and the six closest atoms of the surface layers (making up the extended pyramids of 10 atoms each) was modified. For the case of abrupt junctions, the Al or Ni atoms of only the right pyramid were completely replaced by Pt or Fe, respectively, while for alloy junctions the composition of both extended pyramids was partially modified in a random way. Note that such geometries rely on the assumption that principal scattering processes occur in the narrowest part of the system - at the pyramid-pyramid junction - rather than at the pyramid/electrode interfaces, which seems to be reasonable due to the significantly larger atomic coordination of the latter. These initial structures were subsequently optimized, where the atoms of only two surface layers and the pyramids were allowed to relax until the atomic forces were smaller than 10-4 Ry/bohr.

The (spin-polarized) transport calculations were carried out using PWcond[S5] code (included in QE), the supercell described above was augmented by 3 atomic layers on each side and then attached to two semi-infinite electrodes. The total transmission function was calculated by averaging over a (6x6) in-plane mesh of k-points.

Section 4: Supplementary table of metal hardness

Metals	Ni	Fe	Pt	Al	Au
Mohs scale	4.0	4.0	3.5	2.75	2.5
Vickers hardness (MPa)	638	608	400-549	160-350	188-216

Table S1: Hardness of metals at room temperature, based on Ref. S6.

Section 5: Recognition of Al atoms in atomic chains formed in Al-Pt junctions

While the elongation of Al-Pt atomic junctions does not form atomic chains, it is shown in the main text that following the application of a +1V pulse for 200 μsec (electrons are injected from the Pt electrode), suspended atomic chains can be elongated between the electrode apices during the stretching process of Al-Pt junctions. The lack of a clear set of peaks in the length histogram presented in Figure 4d in the main text indicates structural richness beyond that of atomic chains that contain a single type of atoms. Thus, the presence of Al atoms in the atomic chains can be expected. Here, we provide a clear indication for the presence of Al atoms within the formed atomic chains.

Stretching Al-Al as well as Pt-Pt atomic contacts leads to an increase in the conductance due to an increase in the local density of states at the Fermi energy as a result of changes in the overlap of atomic orbitals[S7-S10]. However, the increase in the conductance of stretched Al atomic scale contacts has a very pronounced and peculiar convex shape, as can be seen in Figure S3a[S7,S11-S13]. For Pt atomic scale contacts, the exact shape of the conductance increase during stretching is less defined, though it has mostly a concave shape as observed in Figure S3b[S2,S10]. Figures S3c-e present typical traces of conductance vs. displacement for Al-Pt that were measured after the application of a +1V pulse. The detection of the mentioned typical convex features at different locations along the traces indicates the presence of Al atoms in the elongated atomic chain.

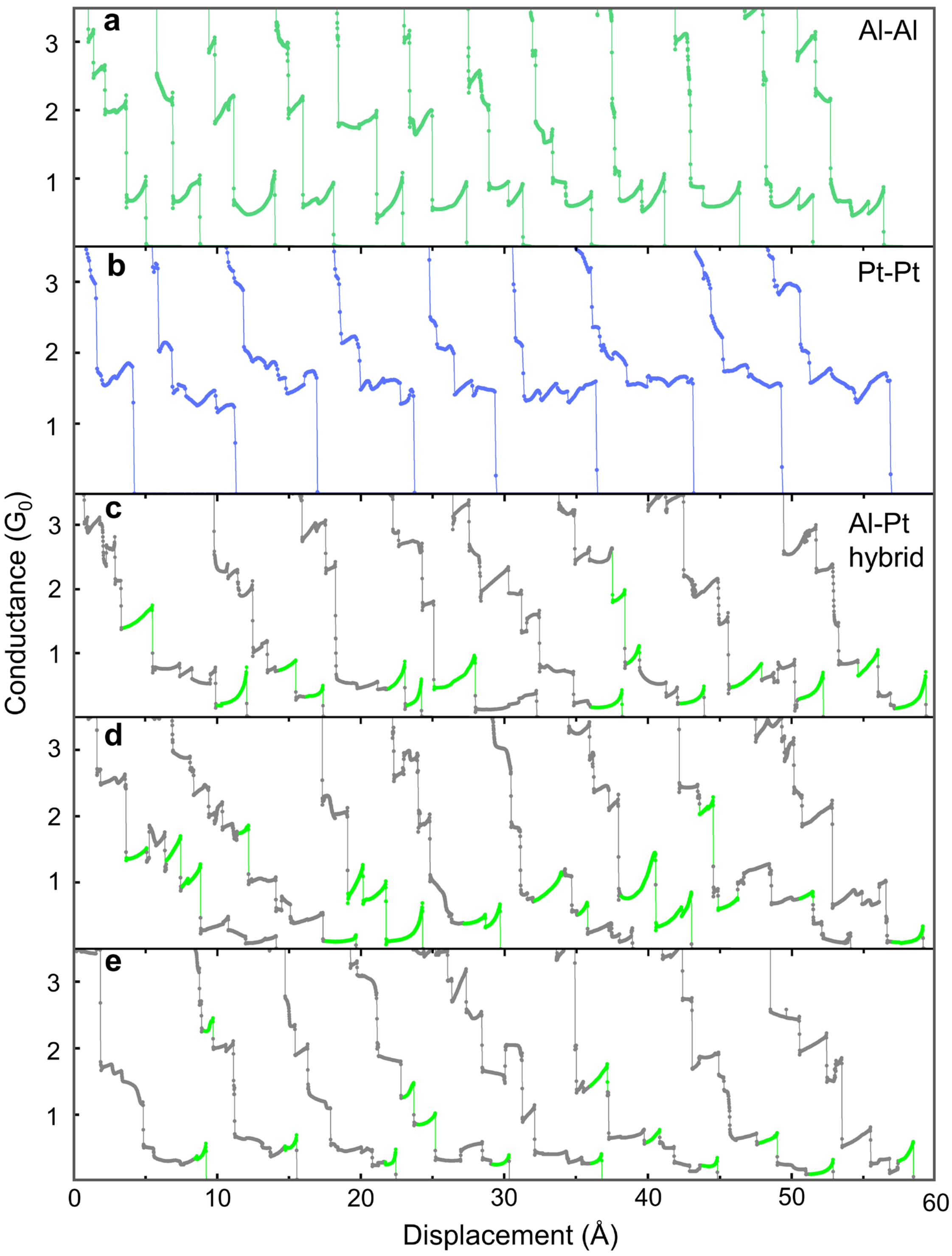

Figure S3: Conductance versus displacement of different junctions. Examples for traces of conductance versus interelectrode displacement for Al-Al junctions (a), Pt-Pt junctions (b), and Al-Pt junctions after the application of a +1V pulse (c-e). Convex features that indicate the presence of Al atoms are marked in (c-e) in green. The measurements were done at an applied voltage of 100 mV.

Section 6: Al-Pt junction response to repeated deformation cycles after pulse application

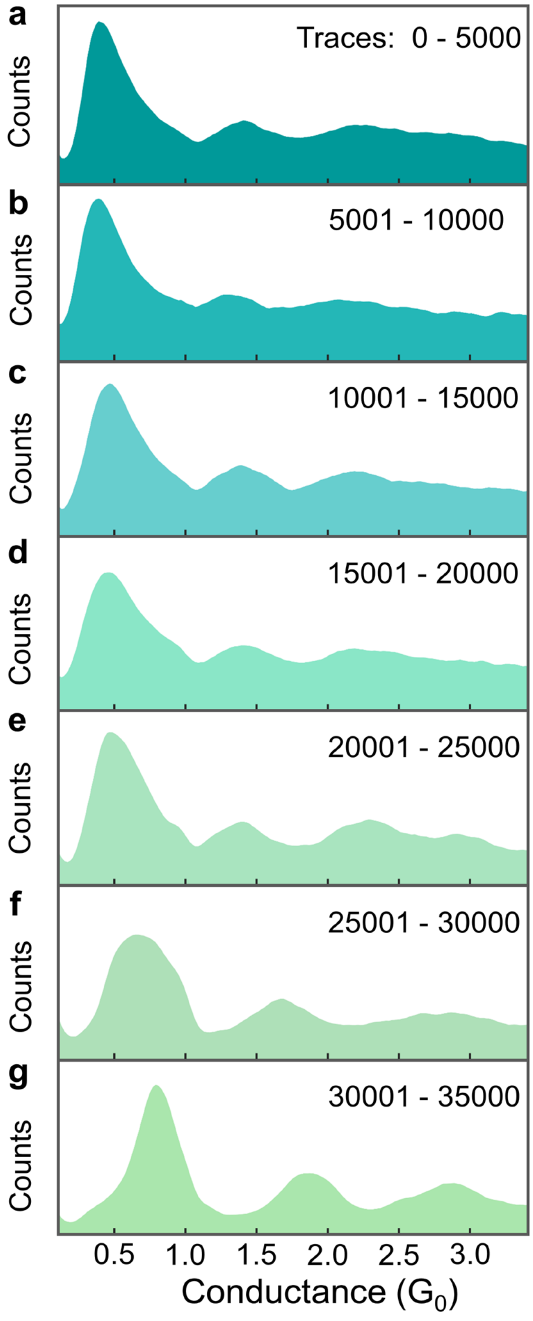

Figure S4: Evolution of conductance histograms of Al-Pt atomic-scale junctions after the application of a positive voltage pulse. The conductance histogram is gradually changing. For example, the main peak at ~0.4 G_0 is shifted to ~0.8 G_0. To achieve a detectable change over 35,000 traces, the two electrodes are squeezed repeatedly to have a contact of ~25 G_0. The total time elapsed between traces number 1 and 35,000 is about 50 min. Each conductance histogram is based on 5,000 conductance versus inter-electrode displacement traces, taken during junction elongation at an applied voltage of 100 mV.

Following an applied voltage pulse to the Al-Pt bimetallic junction with electrons injected from the Pt electrode, a bimetallic structure is formed in the junction. However, in response to repeated mechanical manipulation with high enough squeezing amplitude (tens of G_0), the contact is changing as a function of squeezing events to eventually yield the characteristics of Al-Al junctions, as seen in Figure S4. To preserve an Al-Pt bimetallic contact for a practical timeframe of several hours, one needs to limit the squeezing amplitude of the contacts to a few G_0.

Section 7: Fe-Ni spin valve experiments

Before the application of magnetic fields, the Fe-Ni junctions were repeatedly broken and reformed for thousands of times until stable histograms at 100 mV were obtained as a function of time. Next, a constant magnetic field of +3 Tesla was applied perpendicular to the junction's axis and the junction was broken and reformed for several thousands of times. This procedure promotes initial alignment of magnetization. In the following step, a constant magnetic field of +1 Tesla was applied perpendicular to the junction's axis and several consecutive conductance histograms of 5,000 traces each were collected (e.g., Figure S5a). The magnetic field was chosen to be perpendicular to the junction since in former experiments, where magnetic fields were applied along the junction's axis, we could observe magneto-conductance behavior that can be ascribed to a non-collinear magnetization in the two electrodes due to an easy axis of magnetization perpendicular to the junction's axis. Several consecutive histograms were taken to verify that the histograms do not evolve under magnetic field as a function of time. Overall, we repeated the same procedure for different constant magnetic fields, starting from +1 Tesla and ending at -1 Tesla proceeding with magnetic field steps of 0.1 Tesla (e.g., Figure S5). Minus and plus signs represent magnetic fields in opposite directions. Following the described set of measurements (corresponding to the black data set in Figure 5), we immediately continued to the next set of measurements (corresponding to the red data set in Figure 5), were we repeated the described measurement procedure. However, by advancing from -1 Tesla to +1 Tesla (e.g., Figure S6).

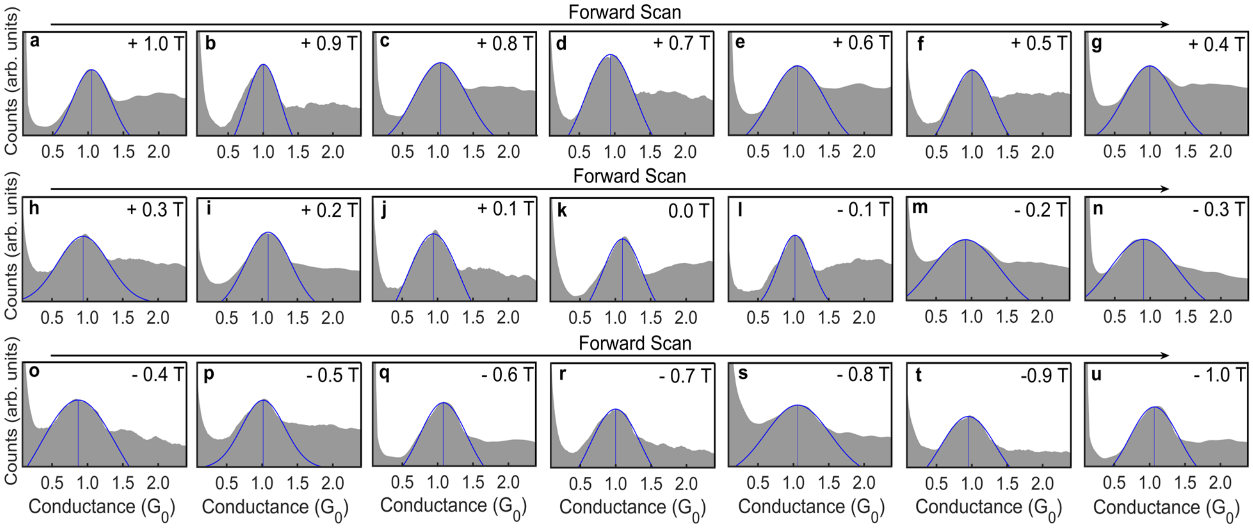

Figure S5: Conductance histograms of Fe-Ni atomic-scale junctions as a function of magnetic field applied perpendicular to the junctions. Gaussian fits and their centers are presented in blue. The extracted value of the most probable conductance by this fitting served to construct the black curve in Figure 5. Specifically, at each magnetic field presented in Figure 5, we took an average of the most probable conductance values, using Gaussian fitting to several different conductance histograms. Here, we present an example for one histogram at each field. Each conductance histogram is based on 5,000 conductance-elongation traces, at a voltage of 100 mV.

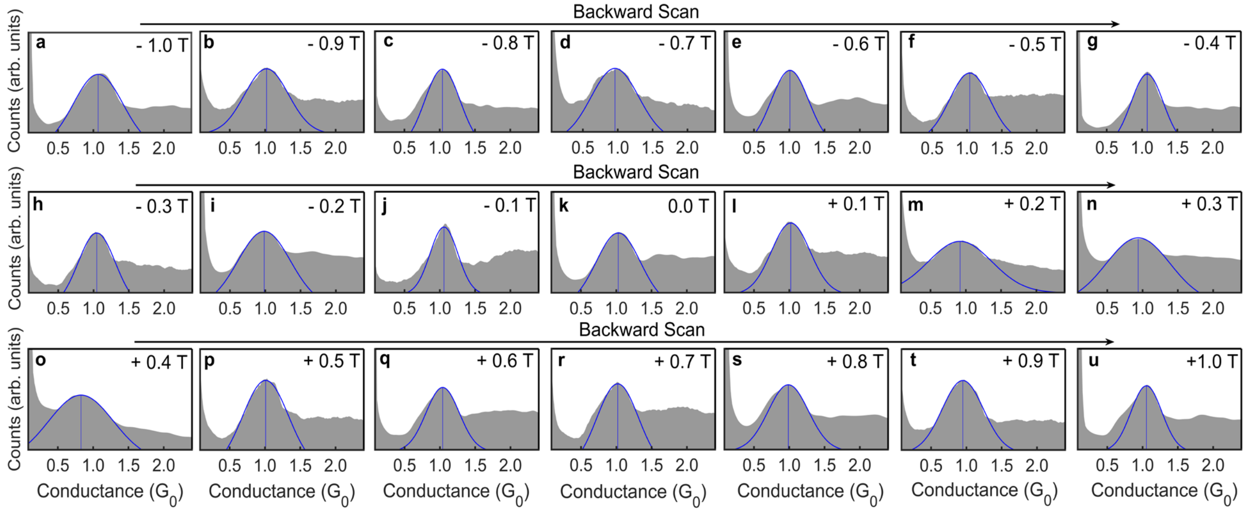

Figure S6: Conductance histograms of Fe-Ni atomic-scale junctions as a function of magnetic field applied perpendicular to the junctions. Gaussian fits and their centers are presented in blue. The extracted value of the most probable conductance by this fitting served to construct the red curve in Figure 5. Specifically, at each magnetic field presented in Figure 5, we took an average of the most probable conductance values, using Gaussian fitting to several different conductance histograms. Here, we present an example for one histogram at each field. Each conductance histogram is based on 5,000 conductance-elongation traces, at a voltage of 100 mV.

Figure S7 shows data from control experiments similar to the spin-valve measurements presented in Figure 5. Here, we repeated the same measurement procedure, however for Ni-Ni and Fe-Fe junctions. As can be seen, we did not detect the typical spin valve "butterfly" behavior. Note that the conductance response to magnetic field for Ni-Ni and somewhat for Fe-Fe is typical to anisotropic magnetoresistance[S14,S15].

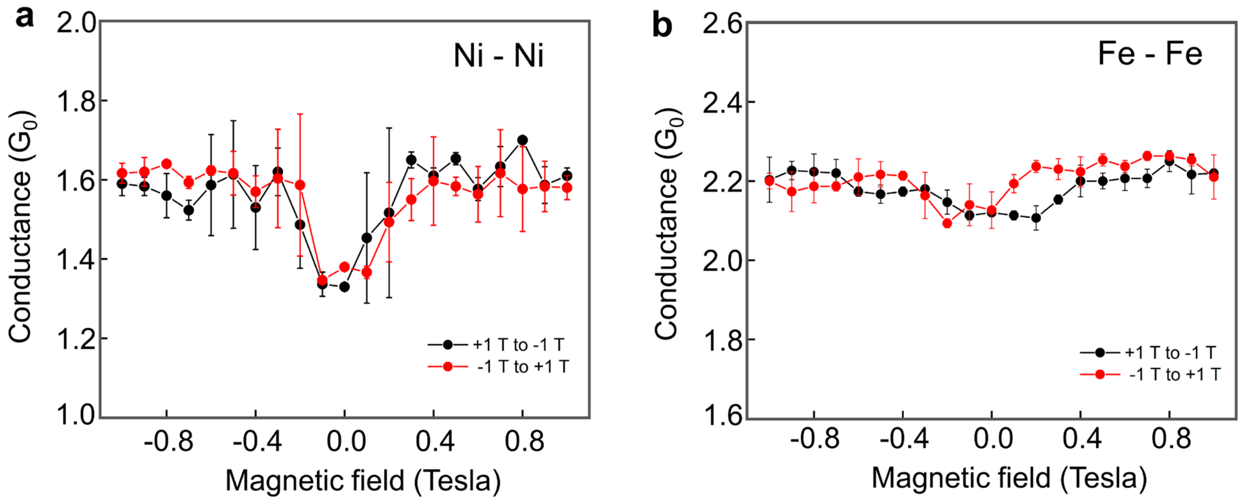

Figure S7: Conductance versus magnetic field for Ni-Ni and Fe-Fe junctions. Most probable conductance of Ni-Ni atomic junctions (a) and Fe-Fe atomic junctions (b), as a function of applied magnetic fields perpendicular to the junction axis (T denotes Tesla). The data at each magnetic field is obtained from several consecutive conductance histograms. Each histogram is based on 10,000 conductance traces measured during junction elongation, at a bias voltage of 100 mV. The error bars provide the standard deviation of the averaged data.

References:

S1. Muller, C. J.; van Ruitenbeek, J. M.; de Jongh, L. J. Experimental observation of the transition from weak link to tunnel junction. *Phys. C (Amsterdam, Neth.)* **1992**, 191, 485–504.

S2. Chakrabarti, S.; Vilan, A.; Deutch, G.; Oz, A.; Hod, O.; Peralta, J. E.; Tal, O. Magnetic control over the fundamental structure of atomic wires. *Nature Commun.* **2022**,13, 1-2 DOI:10.1038/s41467-022-31456-4

S3. Paolo, Giannozzi et al. QUANTUM ESPRESSO: a modular and open-source software project for quantum simulations of materials. *J. Phys. Condens. Matter* **2009**, 21, 395502 DOI:10.1088/0953-8984/21/39/395502

S4. Perdew, J. P.; Burke, K.; Ernzerhof, M. Generalized gradient approximation made simple. *Phys. Rev. Lett.* **1996**, 77, 3865 DOI:https://doi.org/10.1103/PhysRevLett.77.3865

S5. Smogunov, A.; Dal Corso, A.; Tosatti, E. Ballistic conductance of magnetic Co and Ni nanowires with ultrasoft pseudopotentials. *Phys. Rev. B* **2004** 70, 045417 DOI:https://doi.org/10.1103/PhysRevB.70.045417
S6. Samsonov; G. V. Handbook of the Physicochemical Properties of the Elements. Springer, **2012**.
S7. Cuevas, J. C.; Levy Yeyati, A.; Martín-Rodero, A.; Rubio Bollinger, G.; Untiedt, C.; Agraït, N. Evolution of Conducting Channels in Metallic Atomic Contacts under Elastic Deformation. *Phys. Rev. Lett.* **1998**, 81, 2990 DOI:10.1103/PhysRevLett.81.2990
S8. Jelínek, P.; Pérez, R.; Ortega, J.; Flores, F. First-principles simulations of the stretching and final breaking of Al nanowires: Mechanical properties and electrical conductance. *Phys. Rev. B* **2003**, 68, 085403 DOI: 10.1103/PhysRevB.68.085403
S9. García-Suárez, V. M.; Rocha, A. R.; Bailey, S. W.; Lambert, C. J.; Sanvito, S.; Ferrer, J. Conductance Oscillations in Zigzag Platinum Chains. *Phys. Rev. Lett.* **2005**, 95, 256804 DOI:10.1103/PhysRevLett.95.256804
S10. Vardimon, R.; Yelin, T.; Klionsky, M.; Sarkar, S.; Biller, A.; Kronik, L.; Tal, O. Probing the Orbital Origin of Conductance Oscillations in Atomic Chains. *Nano Lett.* **2014**, 14 (6), 2988–2993, DOI: 10.1021/nl4041737
S11. Krans, J. M.; Muller, C. J.; Yanson, I. K.; Govaert, T. C. M.; Hesper, R.; van Ruitenbeek J. M. One-atom point contacts. *Phys. Rev. B* **1993**, 48, 14721– 14724 DOI:10.1103/PhysRevB.48.14721
S12. Scheer, E.; Joyez, P.; Estève, D.; Urbina, C.; Devoret, M. H. Conduction Channel Transmissions of Atomic-Size Aluminum Contacts *Phys. Rev. Lett.* **1997**, 78, 3535– 3538 DOI: 10.1103/PhysRevLett.78.3535
S13. Vardimon, R.; Klionsky, M.; Tal, O. Experimental determination of conduction channels in atomic-scale conductors based on shot noise measurements *Phys. Rev. B* **2013**, 88, 161404 DOI:10.1103/PhysRevB.88.161404
S14. Bolotin, K. I.; Kuemmeth, F.; Ralph, D. C. Anisotropic Magnetoresistance and Anisotropic Tunneling Magnetoresistance due to Quantum Interference in Ferromagnetic Metal Break Junctions, *Phys. Rev. Lett.* **2006**, 97, 127202 DOI:10.1103/PhysRevLett.97.127202
S15. Keane, Z. K.; Yu, L. H.; Natelson, D. Magnetoresistance of atomic-scale electromigrated nickel nanocontacts, Appl. Phys. Lett. 2006, 88, 062514 DOI:10.1063/1.2172232
